# Effects of domestication on biobehavioural profiles: a comparison of domestic guinea pigs and wild cavies from early to late adolescence

**DOI:** 10.1186/1742-9994-11-30

**Published:** 2014-04-09

**Authors:** Benjamin Zipser, Anja Schleking, Sylvia Kaiser, Norbert Sachser

**Affiliations:** 1 Department of Behavioural Biology, University of Münster, Badestrasse 13, 48149 Münster, Germany; 2 Otto Creutzfeldt Center for Cognitive and Behavioral Neuroscience, University of Münster, Münster, Germany

**Keywords:** Biobehavioural profile, Cortisol, Domestication, Emotionality, Social behaviour, Stress

## Abstract

**Introduction:**

Domestication can lead to marked alterations in the biobehavioural profile of a species. Furthermore, during ontogeny, the individual phenotype of an animal can be shaped by the environment in important phases such as adolescence. We investigated differences in biobehavioural profiles between domestic guinea pigs and their ancestor, the wild cavy, over the course of adolescence. At this age, comparisons between the two groups have not been conducted yet. Male guinea pigs and cavies were subjected to a series of tests twice: during the early and late phase of adolescence. We analysed emotional and social behaviours as well as cortisol reactivity and testosterone levels.

**Results:**

Concerning emotional behaviour, cavies were more explorative and showed more anxiety-like behaviour in the open field test and dark-light test. They also were more risk-taking when having to jump off an elevated platform. Regarding social behaviour, cavies showed less social activity towards unfamiliar females and infants. Furthermore, while guinea pigs and cavies did not differ in basal cortisol levels, cavies showed distinctly higher and prolonged cortisol responses when exposed to an unfamiliar environment. Cavies also had lower basal testosterone titres. No substantial changes in biobehavioural profiles were revealed over the course of adolescence in both groups.

**Conclusions:**

Domestication led to a substantial shift in the biobehavioural profile of the guinea pig regarding all investigated domains in early and late adolescence. Hence, the differentiation between guinea pigs and cavies emerges early in ontogeny, well before the attainment of sexual maturity. The young individuals already show adaptations that reflect the differences between the natural habitat of cavies and the man-made housing conditions guinea pigs are exposed to. Higher levels of exploration and risk-taking and lower levels of anxiety-like behaviour are necessary for cavies in order to cope with their challenging environment. Their high cortisol reactivity can be interpreted as an energy provisioning mechanism that is needed to meet these demands. By contrast, guinea pigs are adapted to a less challenging environment with much higher population densities. Hence, their biobehavioural profile is characterised by higher levels of social activity and lower levels of exploration, risk-taking, and cortisol reactivity.

## Introduction

Domestication - as an evolutionary process - is based on the removal of some natural selection pressures, new natural selection by artificial environments, and artificial selection by humans [1-4]. During their domestication, originally wild animals undergo distinct changes in their biobehavioural profile regarding morphology, physiology, and behaviour [2,5-8]. On a behavioural level, domesticated animals are frequently characterised by reduced aggression, attentiveness, and flight behaviours as well as by an increase in their sexual and courtship behaviour [1-4,8,9].

For example, since its domestication from the wild cavy (*Cavia aperea*), approximately 3000 – 6000 years ago in the highlands of South America [10-13], the biobehavioural profile of the domestic guinea pig (*Cavia aperea* f. *porcellus*) has been altered considerably. It is well known that adult guinea pigs show more sociopositive and courtship behaviour, less aggression, exploration, and orientation behaviour and also differ from their wild ancestor regarding their learning abilities [4,9,14]. Furthermore, guinea pigs show a significantly lower cortisol response when exposed to an unfamiliar environment as well as higher basal testosterone titres [4].

Of course, the biobehavioural profiles of wild and domesticated animals are not only influenced by their respective evolutionary heritage. During ontogeny, important events such as the attainment of sexual maturity also have vast and long lasting impacts on the phenotype of an animal [15-17]. Sexual maturity is reached within the gradual transition from infancy to adulthood. This important phase of ontogeny - called adolescence - comprises extensive alterations in anatomy, endocrine systems, neuronal circuits and behaviour [15,17-19]. Guinea pigs are a prime example showing how crucial the phase of adolescence can be for the development of an individual since behaviour as well as endocrine profiles are heavily influenced by the social circumstances the individual is exposed to during this period (reviews: [17,20,21]). Males living with a single female are characterised by a heightened hypothalamic–pituitary–adrenal (HPA) activity, reduced plasma testosterone titres and elevated levels of aggression [22-24]. By contrast, males living in large mixed-sex colonies show the exact opposite pattern.

Despite a growing literature on domestication in general, there are only few studies directly comparing domestic animals to their wild form. The guinea pig and its wild ancestor, the cavy, are among the examples that have been thoroughly studied in this way [4,9]; (see also dogs vs. wolves [25-27]). However, only adult animals have been compared to date and it is not known if the differences found in adults are already present in earlier phases of ontogeny. Hence, the aim of this study was to compare the biobehavioural profiles of male guinea pigs and wild cavies over the course of adolescence. For this purpose, we exposed male guinea pigs and male cavies to a testing regimen that has recently proven valuable to characterise a broad spectrum of biobehavioural traits in guinea pigs [28]. This series of tests investigates the two behavioural domains emotionality and social behaviour as well as the animals’ cortisol reactivity to the psychological stressor of being exposed to a novel, unfamiliar environment as a third domain. In the present study, we additionally evaluated basal testosterone levels since testosterone plays a crucial role in the maturation process of males. Animals were exposed to all tests twice: once during the early phase of adolescence (around 50 days of age) and a second time during the late phase of adolescence (around 120 days of age). These testing phases are suitable since sexual maturity occurs approximately midway between them (around 75 days of age) in male guinea pigs and cavies [29,30]. We hypothesise that distinct differences exist in the biobehavioural profiles of guinea pigs and cavies and that the profiles of both groups are altered when the animals reach sexual maturity.

## Results

In the following, all results are based on the sample sizes N_domestic_ = 10 and N_wild_ = 8 if not stated otherwise.

### Emotionality

**Figure 1:**
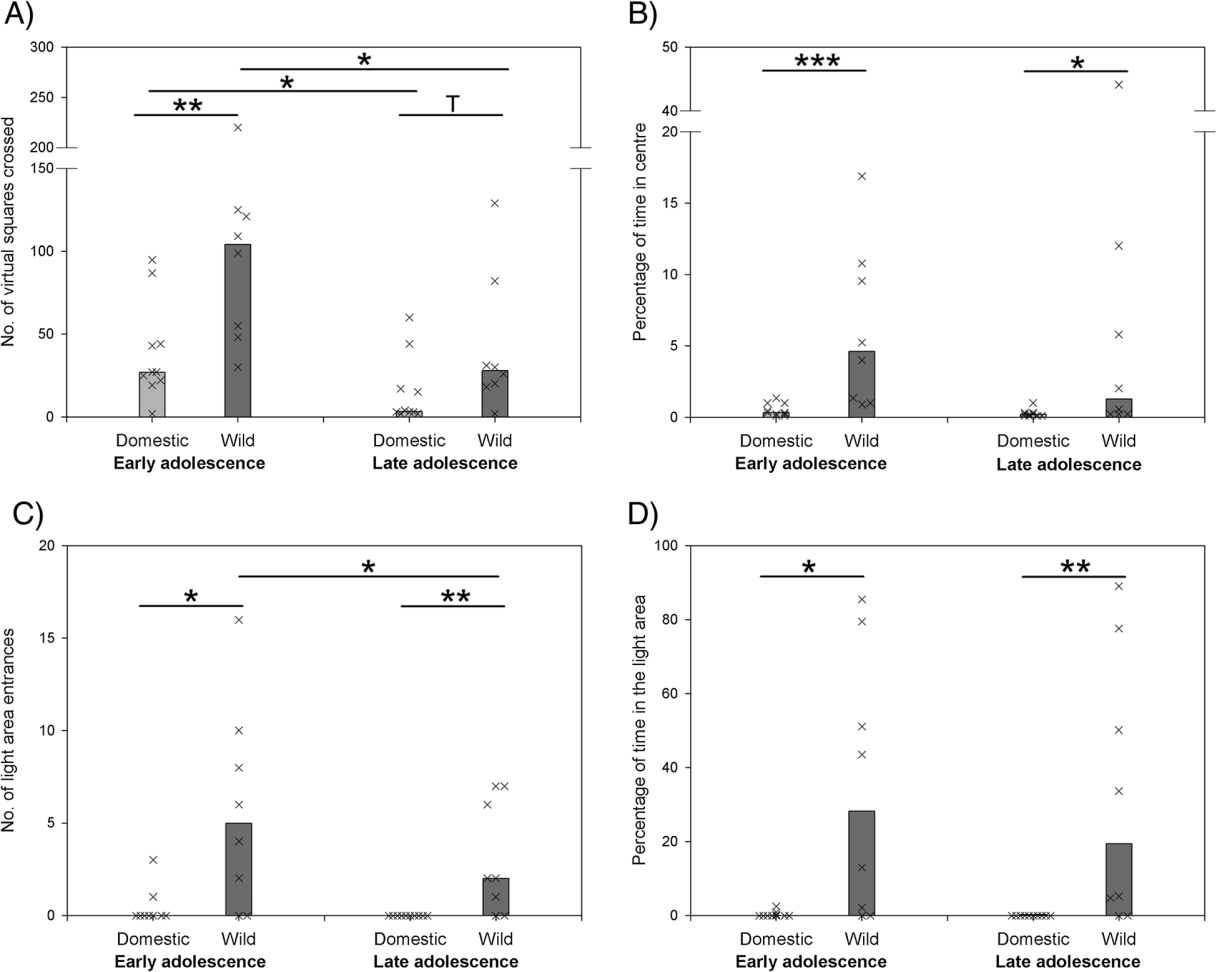
**Behaviour of domestic guinea pigs and wild cavies in two tests on emotional behaviour during early and late adolescence. (A)**: number of virtual squares crossed in an open field test. **(B)**: percentage of time spent in the centre of an open field. **(C)**: number of entrances into the light area of a dark-light test. **(D)**: percentage of time spent in the light area of a dark-light test. Bar charts represent medians and “X”s represent single individuals. N_Domestic_ = 10, N_Wild_ = 8; T = p ≤ 0.1; * = p ≤ 0.05; ** = p ≤ 0.01; *** = p ≤ 0.001.

Regarding emotional behaviour, distinct differences were revealed between guinea pigs and wild cavies during both, early and late adolescence. Wild cavies were generally more explorative as was shown in the open field and dark-light test. Cavies crossed significantly more virtual squares in the open field during early adolescence (U = 8.0, p ≤ 0.003; Figure 1A) and showed a trend to do so in late adolescence (U = 19.0, p ≤ 0.066; Figure 1A). Furthermore, cavies emerged more often from the dark compartment of the dark-light test and thus entered the light area more often in both phases of adolescence (early adolescence: U = 14.0, p ≤ 0.012; late adolescence: U = 10.0, p ≤ 0.002; Figure 1C). Wild cavies also showed less anxiety-like behaviour in the open field and the dark-light test. That is, cavies entered the less protected centre of the open field longer in both phases of adolescence (early adolescence: U = 5.5, p ≤ 0.001; late adolescence: U = 14.5, p ≤ 0.021; Figure 1B). Furthermore, in the dark-light test, cavies entered the coverless light area earlier (early adolescence: median domestic vs. wild = 900 s vs. 128.5 s, U = 16.0, p ≤ 0.023; late adolescence: median domestic vs. wild = 900 s vs. 318 s, U = 10.0, p ≤ 0.002). They also spent more time in the light area (early adolescence: U = 14.0, p ≤ 0.012; late adolescence: U = 10.0, p ≤ 0.002; Figure 1D). Finally, regarding risk-taking in the step down test, cavies jumped earlier from the elevated platform in late adolescence (median domestic vs. wild = 900 s vs. 350 s, U = 21.5, p ≤ 0.043). No such difference was detected during early adolescence (median domestic vs. wild = 900 s vs. 552 s, U = 28.0, p ≤ 0.238).

Over the course of adolescence, exploration decreased in both, guinea pigs (Z = -2.09, p ≤ 0.037) and cavies (Z = -2.1, p ≤ 0.039) in the open field (Figure 1A). Moreover, in the dark-light test, exploration decreased in cavies regarding *number of light area entrances* (Z = -2.13, p ≤ 0.047; Figure 1C) but not in guinea pigs (Z = -1.63, p ≤ 0.25; Figure 1C). No significant changes over the course of adolescence were found regarding all other parameters of emotionality.

### Social behaviour

No differences were found between guinea pigs and cavies concerning the latencies to approach the unfamiliar interaction partners in the two tests on social behaviour. In neither of the two phases of adolescence, guinea pigs and cavies differed in the *latency to approach the unfamiliar infant* in the male vs. infant interaction test (early adolescence: median domestic vs. wild = 146 s vs. 88.5 s, U = 31.5, p ≤ 0.475; late adolescence: median domestic vs. wild = 116 s vs. 36 s, U = 24.0, p ≤ 0.167). Furthermore, there was no difference in the *latency to show courtship behaviour towards the unfamiliar female* in the male vs. female interaction test (early adolescence: N_wild_ = 5, median domestic vs. wild = 129.5 s vs. 222 s, U = 13.5, p ≤ 0.172; late adolescence: median domestic vs. wild = 312 s vs. 549 s, U = 31.5, p ≤ 0.47). Guinea pigs clearly preferred the cage with the infant in it over the empty control cage in both phases of adolescence (early adolescence: median of visits infant vs. empty = 13.5 vs. 4, Z = -2.67, p ≤ 0.004; late adolescence: median of visits infant vs. empty = 15.5 vs. 3, Z = -2.8, p ≤ 0.002). Wild cavies only showed a trend for preference of the infant cage in early adolescence (median of visits infant vs. empty = 14.5 vs. 4.5, Z = -1.82, p ≤ 0.078). But they clearly preferred the infant cage in late adolescence (median of visits infant vs. empty = 16.5 vs. 3.5, Z = -2.52, p ≤ 0.008). Hence, both groups were more interested in the cage containing the interaction partner than in the empty control cage.

**Figure 2:**
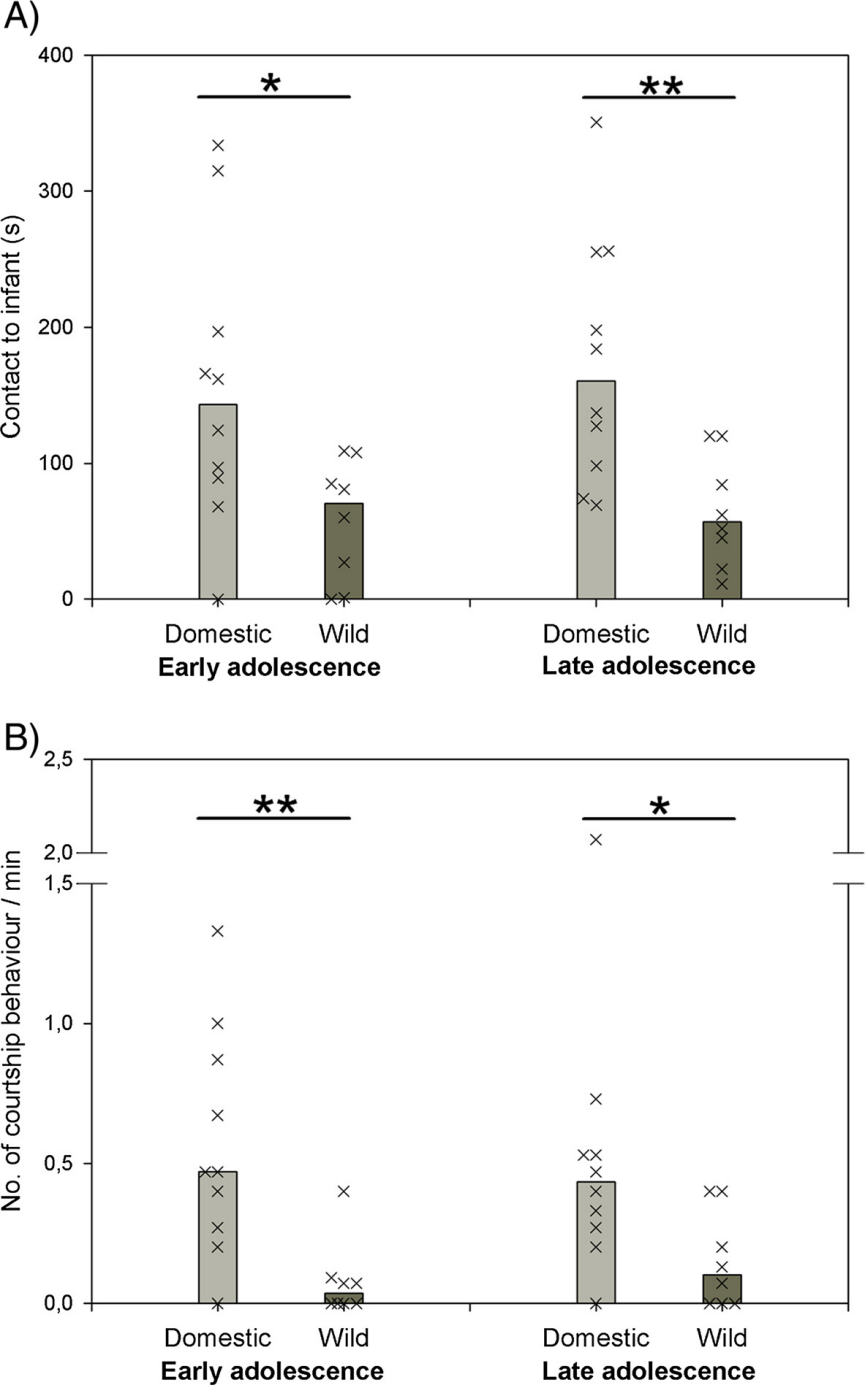
**Behaviour of domestic guinea pigs and wild cavies in two tests on social behaviour during early and late adolescence. (A)**: time spent in contact to an unfamiliar infant in a male vs. infant interaction test. **(B)**: frequency of courtship behaviour shown towards an unfamiliar female in a male vs. female interaction test. Bar charts represent medians and “X”s represent single individuals. N_Domestic_ = 10, N_Wild_ = 8; * = p ≤ 0.05; ** = p ≤ 0.01.

There was, however, a substantial difference regarding the amount of social interactions guinea pigs and cavies directed towards their interaction partners in the social tests. In the male vs. infant interaction test, guinea pigs spent significantly more time in contact with the unfamiliar infant than cavies in both phases of adolescence (early adolescence: U = 15.5, p ≤ 0.027; late adolescence: U = 8.0, p ≤ 0.003; Figure 2A). Guinea pigs also showed more courtship behaviour towards the unfamiliar female in the male vs. female interaction test in both phases of adolescence (early adolescence: U = 8.5, p ≤ 0.003; late adolescence: U = 14.0, p ≤ 0.018; Figure 2B). Regarding changes of social behaviour over the course of adolescence, the *latency to approach the unfamiliar infant* did not change in guinea pigs (median 146 s vs. 116 s, Z = -0.561, p ≤ 0.625) and cavies (median 88.5 s vs. 36 s, Z = -0.841, p ≤ 0.438). The same was true for *duration of infant contact* in guinea pigs (Z = -0.306, p ≤ 0.789) and cavies (Z = 0.0, p ≤ 0.99), Figure 2A. In the male vs. female interaction test the *latency to show courtship behaviour towards the unfamiliar female* did not change in guinea pigs (median 129.5 s vs. 312 s, Z = -0.357, p ≤ 0.77) and cavies (N_wild_ = 5, median 222 s vs. 549, Z = -0.135, p ≤ 0.99). This was also the case concerning *frequency of courtship behaviour* in guinea pigs (Z = -0.237, p ≤ 0.848) and cavies (Z = -1.214, p ≤ 0.313), Figure 2B.

### Stress reactivity

**Figure 3:**
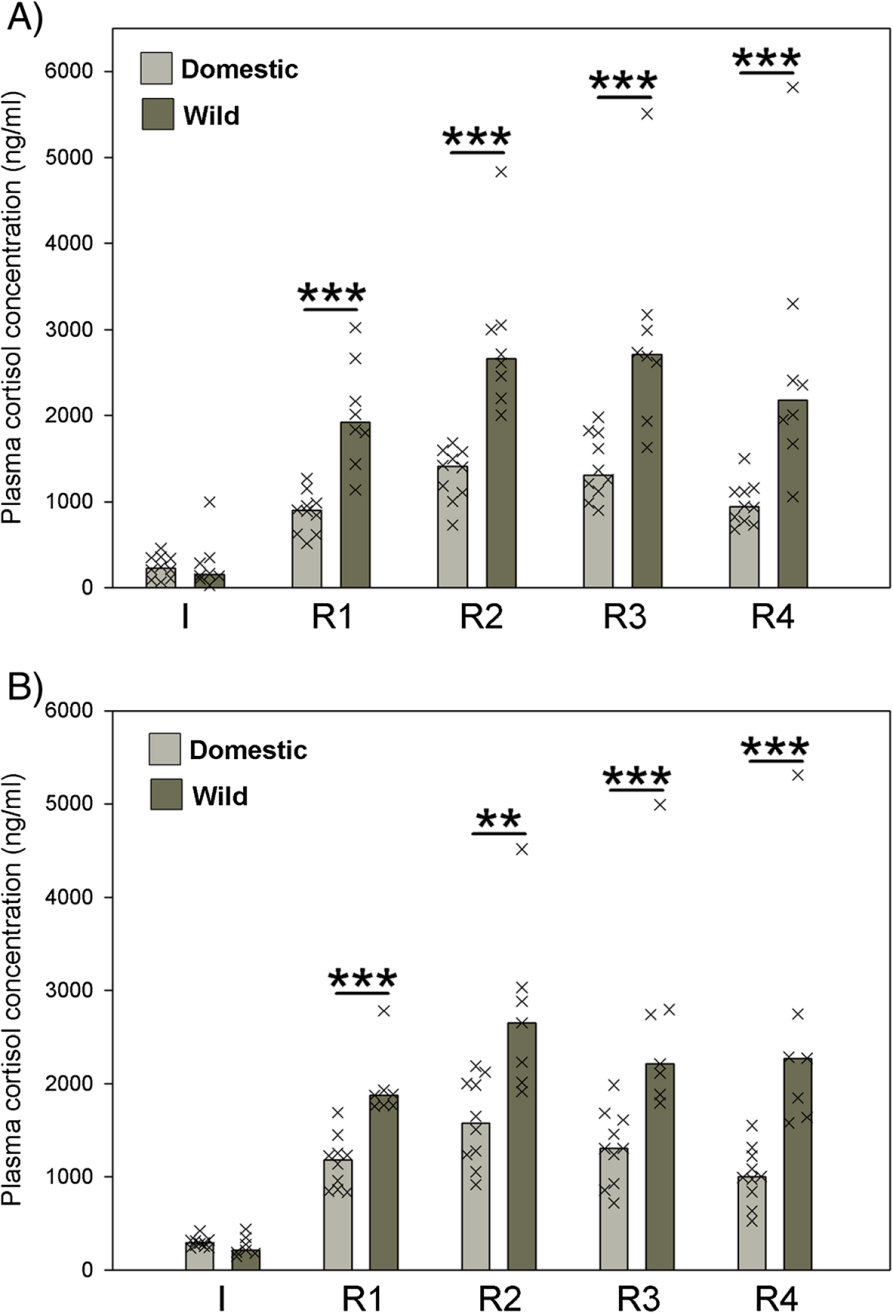
**Plasma cortisol response (ng/ml) in a stress reactivity test: initial values (I) and reaction values (R 1-4) of domestic guinea pigs and wild cavies during early (A) and late (B) adolescence.** Bar charts represent medians and “X”s represent single individuals. N_domestic_ = 10, N_wild_ = 8 (early adolescence) and N_wild_ = 7 (late adolescence); ** = p ≤ 0.01; *** = p ≤ 0.001. Comparison of guinea pig and cavy values was done with Mann-Whitney-U tests whereas development of cortisol values over time within guinea pigs and cavies was analysed with Friedman-tests and Kendall’s W for effect sizes. Friedman tests revealed a significant increase in cortisol values from I to the reaction values in both groups during both phases of adolescence. Guinea pigs: χ^2^ = 35.1, df = 4, p ≤ 0.001, W: 0.878 (early adolescence); χ^2^ = 34.96, df = 4, p ≤ 0.001, W: 0.874 (late adolescence). Cavies: χ^2^ = 21.8, df = 4, p ≤ 0.001, W: 0.681 (early adolescence); χ^2^ = 20.1, df = 4, p ≤ 0.001, W: 0.718 (late adolescence). p ≤ 0.001 for all Kendall W tests.

Both, guinea pigs and cavies, reacted with enduring elevation of plasma cortisol (C) titres when exposed to the novel enclosure in the stress reactivity test during early adolescence as well as late adolescence as revealed by Friedman tests and Kendall’s W for effect sizes (guinea pigs, early adolescence: χ^2^ = 35.1, df = 4, p ≤ 0.001, W: 0.878; guinea pigs, late adolescence: χ^2^ = 34.96, df = 4, p ≤ 0.001, W: 0.874; cavies, early adolescence: χ^2^ = 21.8, df = 4, p ≤ 0.001, W: 0.681; cavies, late adolescence: n = 7, χ^2^ = 20.1, df = 4, p ≤ 0.001, W: 0.718; p ≤ 0.001 for all Kendall W tests, Figure 3). Hence, being transferred from the familiar home enclosure to a novel, unfamiliar environment reliably induced a cortisol response in both groups.

However, guinea pigs and cavies did not differ in their initial C values (before transfer to the novel enclosure) during both phases of adolescence (early adolescence: U = 34.5, p ≤ 0.65; late adolescence: U = 23.0, p ≤ 0.27; Figure 3). By contrast, guinea pigs and cavies differed considerably regarding their reaction values (R1-R4, when exposed to the novel enclosure) during both phases of adolescence (Figure 3). Wild cavies always showed higher C reaction values than guinea pigs (early adolescence: R1, U = 2.0, p ≤ 0.001; R2, U = 0.0, p ≤ 0.001; R3, U = 0.0, p ≤ 0.001; R4, U = 0.0, p ≤ 0.001; late adolescence: R1, U = 0.0, p ≤ 0.001; R2, U = 6.0, p ≤ 0.003; R3, U = 2.0, p ≤ 0.001; R4, U = 0.0, p ≤ 0.001; Figure 3). The same held true when comparing the increase from initial values (I) to the respective reaction values and the increase from I to the maximal C responses (Table 1).

**Table 1:** Absolute increase of plasma cortisol values in a stress reactivity test

		**Early adolescence**	**Late adolescence**
**R1-I (ng/ml)**	Median domestic	586.5	872
	Median wild	1683	1609
	U	0	0
	p	**0.001**	**0.001**
**R2-I (ng/ml)**	Median domestic	1059.5	1308,5
	Median wild	2438	2208
	U	0	6
	p	**0.001**	**0.001**
**R3-I (ng/ml)**	Median domestic	1052	1056
	Median wild	2563	1836
	U	4	2
	p	**0.001**	**0.001**
**R4-I (ng/ml)**	Median domestic	717	714,5
	Median wild	1927	1843
	U	2	0
	p	**0.001**	**0.001**
**MAX-I (ng/ml)**	Median domestic	1133.5	1436.5
	Median wild	2574	2208
	U	0	0
	p	**0.001**	**0.001**

Domestic guinea pigs and wild cavies were transferred from their familiar home enclosure to a novel enclosure for four hours. The absolute increase from initial values (I, in home enclosure) to values after 1 h (R1), 2 h (R2), 3 h (R3), and 4 h (R4) in the novel enclosure as well as from I to the maximal response value (MAX, maximum value of R1-R4) are shown. The responses of guinea pigs and cavies were compared during early and late adolescence. N_domestic_ = 10, N_wild_ early = 8, N_wild_ late = 7. Significant p-values are highlighted in boldface.

Regarding changes of C reactivity over the course of adolescence only one significant difference was detected: R1 in guinea pigs increased significantly during adolescence (median 228.5 vs. 291.5, Z = -2.19, p ≤ 0.027). All other C values, as well as the maximal increase in C in guinea pigs and cavies did not change significantly over the course of adolescence.

### Testosterone levels

**Figure 4:**
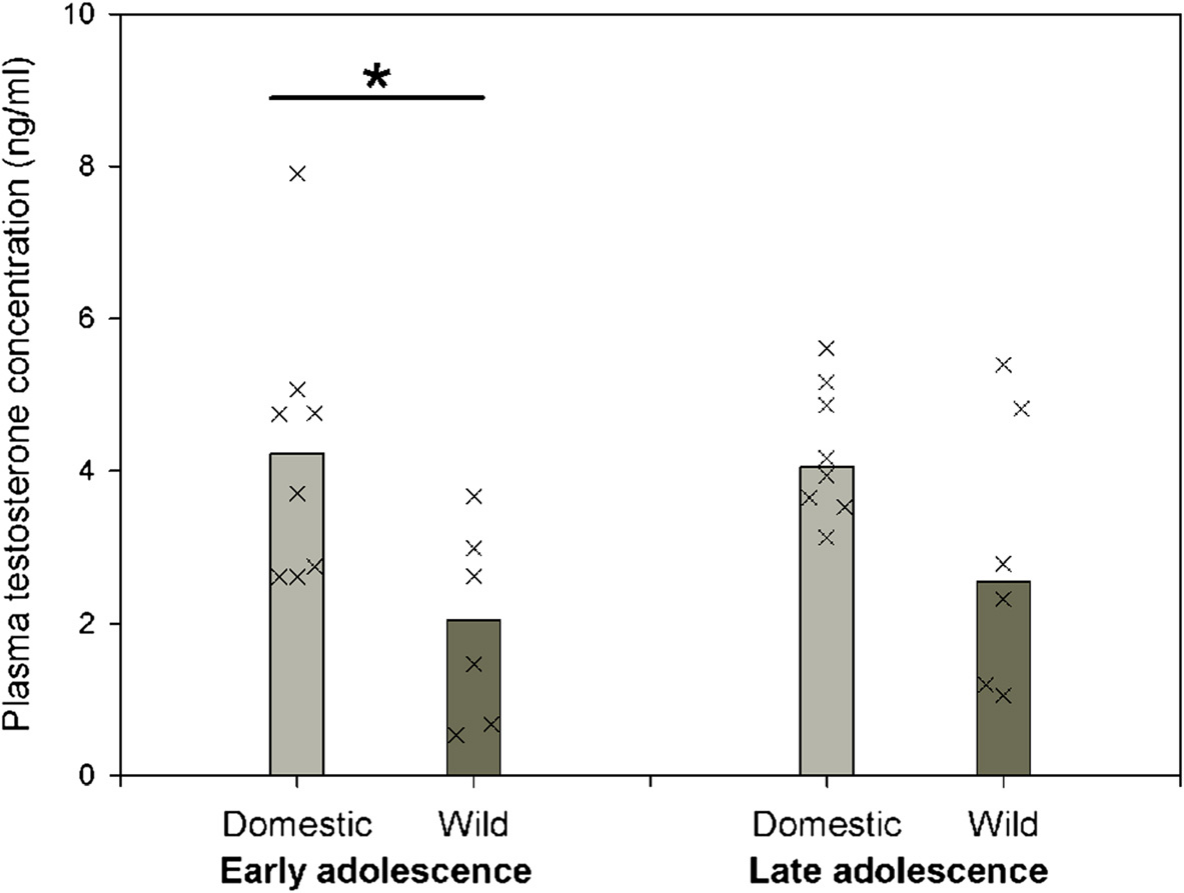
**Basal plasma testosterone levels (ng/ml) of domestic guinea pigs and wild cavies during early and late adolescence.** Bar charts represent medians and “X”s represent single individuals. N_Domestic_ = 8, N_Wild_ = 6; * = p ≤ 0.05.

Basal plasma testosterone (T) levels clearly differed between guinea pigs and cavies during early adolescence. Guinea pigs had significantly higher T levels (N_domestic_ = 8, N_wild_ = 6, U = 8, p ≤ 0.039, Figure 4). Median T levels were higher in guinea pigs during late adolescence as well, however not significantly so (N_domestic_ = 8, N_wild_ = 6, U = 12, p ≤ 0.121, Figure 4). No significant changes of T levels over the course of adolescence were detected in guinea pigs and cavies (Figure 4).

## Discussion

The present study compared domestic guinea pigs and their wild ancestor, the cavy, regarding their biobehavioural profiles. Three domains were investigated: emotionality, social behaviour, and cortisol reactivity. In addition, basal testosterone titres were compared. Furthermore, we investigated the development of these traits over the course of adolescence in both groups. While no major changes in biobehavioural profiles over the course of adolescence were revealed, guinea pigs and cavies differed considerably in the majority of all behavioural and endocrinological parameters during both, early and late adolescence. From an ontogenetic perspective this implies that the differences between the two groups are already present early in life, well before the attainment of sexual maturity. It is, however, still unclear how these differences are caused and at which ontogenetic phase they emerge. In principle, genetic differences, impacts of the early pre- and postnatal environment, or an interaction of both could be underlying the differentiation in biobehavioural profiles. Indeed, maternal effects in these early phases of life are a well-known phenomenon that can shape the biobehavioural profiles of guinea pigs and cavies (reviewed in [17,21]). Future experiments comparing both groups during this period could shed more light on the mechanisms driving the ontogenetic differentiation between the domestic and wild form of the species.

### Contrasting biobehavioural profiles in guinea pigs and wild cavies

#### Emotionality

Guinea pigs and cavies behaved significantly different regarding all parameters of emotionality. Guinea pigs were less explorative and showed more anxiety-like behaviour in the open field and dark-light test. Furthermore, they showed less risk-taking behaviour in the step down test in late adolescence. A comparable difference regarding exploration has also been found in adult animals in a paradigm that did not force the subjects into the test situation but was based on voluntary exploration from the security of a familiar enclosure [9]. As in the dark-light test of this study – in which most guinea pigs did not leave the dark box - most guinea pigs did not leave the familiar enclosure at all.

From an evolutionary standpoint, extensive exploration is crucial for wild animals like cavies in order to obtain access to vital resources such as water, food, shelter, and mates. But exploring the environment for these resources is risky, challenging and at times dangerous. In the natural habitat of wild cavies, predation can be so severe that mortality rates of up to 50% can be observed in a five month period [31]. In such a scenario, animals are forced to overcome their tendency to avoid novelty [32] and thus exploration and a certain amount of risk-taking are necessary. In contrast to this situation in the wild, domestication is characterised by a removal of dangerous and challenging environmental factors [3]. In man-made housing systems, guinea pigs are usually provided with all relevant resources and hence the selection pressure for high levels of exploration and risk-taking was removed. The humans who kept cavies at their homes probably chose (or were only able to catch and keep) animals that were less prone to flee the artificial housing in their homes – that is, they selected animals with less exploratory and risk-taking tendencies.

#### Social behaviour

Clear differences between guinea pigs and cavies were also found regarding social behaviour. In both, the male vs. infant interaction test and male vs. female interaction test, guinea pigs engaged in more interactions with the partner animal. However, the latencies to approach the interaction partners did not differ between guinea pigs and cavies in both tests. These results show that both groups were interested in the social stimuli but after initially exploring the novel situation, guinea pigs remained in contact with the partner to a greater extent. Similar findings have been made in studies comparing adult guinea pigs and cavies [4,9]. In these experiments, guinea pig males showed more sociopositive behaviour and courtship behaviour towards their familiar group mates. In the present study, we investigated the animals’ reaction to an unfamiliar animal in a novel situation. Our results thus represent the animals’ tendency to socially explore new interaction partners. Furthermore, it is likely that the male vs. infant interaction test is not influenced by sexual or aggressive motivations since an infant of the same sex was used as an interaction partner. Hence, we conclude from the male vs. infant and male vs. female interaction test, that guinea pigs have both, a tendency to show more courtship behaviour as well as more social interaction in general. Such traits are typical for domesticated animals in which higher degrees of agreeableness and sexual behaviour as well as lower levels of aggression are common [2-4,9,33]. One of the major factors driving this shift in biobehavioural profiles from wild cavies to guinea pigs during domestication was the tremendous increase in population densities. While cavies live in large home ranges from 200 m^2^ up to 1000 m^2^[31,34], guinea pigs can be kept in groups of up to 20 animals in 6 m^2^ enclosures without any problems [35]. This certainly forced the first breeders of wild cavies to choose and select for the most agreeable and least aggressive individuals since otherwise it would not have been possible to keep a reasonably large stock of animals in captivity.

#### Endocrine parameters

Guinea pigs and cavies differed markedly regarding their endocrine profiles. While initial C levels of guinea pigs and cavies in their familiar home enclosure were not significantly different in both, early and late adolescence, all four response values in the stress reactivity test were significantly higher in cavies in these two phases.

These differences in the endocrinological reaction to a challenging situation most likely have their foundation in the differences between the natural habitat cavies are adapted to and the man-made housing conditions guinea pigs were adapted to in the process of domestication. As a wild species, cavies evolved in a highly challenging environment in which resources are limited and have to be competed for in order to survive and reproduce [31,34]. As coping in such demanding circumstances is energy expensive, cavies require appropriate physiological adaptations to provide the necessary energy quickly when the situation calls for it [36]. One of the main functions of the HPA-axis as part of the stress system is exactly that: to supply the organism with the necessary energy when coping with challenges. From this point of view, a high reactivity of the stress axes must not necessarily represent a detrimental impact on animals due to increased stress [37,38]. It is rather an energy provisioning mechanism that is necessary for a wild animal in order to deal with its everyday life. Hence, we suppose that the high C reactivity found in cavies represents an adaptation to their demanding life in the wild which can still be observed under laboratory conditions. One can assume that the C reactivity of the cavies investigated in the present study represents a genuine characteristic of the wild living cavy. No differences regarding behaviour and C reactivity can be found when comparing cavies bred in the laboratory over 30 generations to wild trapped animals (and their first laboratory reared offspring) [9]. In contrast to the natural habitat of cavies, man-made housing systems are considerably less challenging since they provide the animals with virtually all relevant resources such as food, shelter and mates. Consequently, the energy needed to engage in everyday activities is also considerably lower for guinea pigs, which renders a high reactivity of the C response less valuable.

Concerning basal plasma testosterone levels, guinea pigs had higher values in early as well as late adolescence (though not significantly so concerning the latter). Additionally, guinea pigs showed increased levels of courtship behaviour. Both differences are also found when comparing fully adult animals [4] and a correlation between increased levels of testosterone and hypertrophy of courtship and sexual behaviour are frequently found in domesticated animals [3,4,8,39]. Since social interactions including courtship behaviour can result in increased testosterone titres [40-42] there might be a causal relationship between increased amounts of courtship behaviour and higher levels of testosterone in domesticated animals, including guinea pigs.

Our results can also be interpreted in the light of a recently suggested model on the adaptive shaping of biobehavioural profiles over the course of adolescence [17]. This model states that the more social interactions (especially agonistic and sexual/courtship) the individual is involved in during adolescence, the higher its T levels are, which is in accordance with the challenge hypothesis [42,43]. The T levels in turn organise the C reactivity of an individual with higher T levels resulting in lower levels of C reactivity [23]. Since high acute C reactivity can trigger aggression [44-46] the model can also explain differences in agonistic behaviour between animals.

Applying this model to the findings of the present study could explain some of the behavioural differences found between domestic guinea pigs and cavies as well as the physiological mechanisms underlying these differences. We showed that adolescent guinea pigs engage in more social activity and have higher T levels than wild cavies. These differences persist into adulthood [4,9]. Although we did not monitor social activities in the male-male housing groups one can still assume that guinea pigs were also more socially active than cavies in their home enclosure since this is the case in adult animals [4,9]. High levels of social interaction in adolescent guinea pigs might result in the higher levels of testosterone found in our study. The high T levels might in turn organise the low cortisol responsiveness we found in adolescent guinea pigs. Since low acute C reactivity results in low levels of aggression [44-46] these differences in endocrine fine-tuning might be the mechanistic basis for the low levels of male aggression found in guinea pigs in comparison to their wild ancestor [4,9]. Thus, the same mechanisms shaping the biobehavioural profiles of guinea pigs exposed to varying degrees of social interaction during adolescence [17,21] could also be involved in the differentiation of the biobehavioural profiles of guinea pigs versus cavies.

### Changes in biobehavioural profiles over the course of adolescence

Hardly any changes of behavioural traits as well as endocrine parameters were found over the course of adolescence in both, guinea pigs and cavies. At first glance, the fact that no changes of T levels occurred in both groups might come as a surprise, especially since sexual maturity is reached in cavies and guinea pigs between early and late adolescence. The development of plasma T titres is, however, highly dependent on the social housing conditions an animal lives in. For example, while colony housed guinea pig males show a significant increase of T levels over the course of adolescence, individually housed animals and animals housed with a single female do not [47,48].

Of the total of 16 variables we measured here, only one showed a significant decrease in both groups: *number of virtual squares crossed* in the open field. Additionally, the *number of light area entrances* in the dark-light test decreased in wild cavies and the first reaction value in the stress reactivity test increased in guinea pigs. Since both behavioural variables that changed could be influenced by habituation effects and no changes were detected regarding all other C variables, these results are hard to interpret. But since the vast majority of variables did not change over the course of adolescence our overall conclusion is that emotionality, social behaviour, C reactivity as well as basal plasma T levels seem to be relatively unaffected during adolescence when no challenging external influences act upon the animals. Of course, other traits not investigated in this study - such as aggression - might be influenced. But still our findings endorse the idea that it is not adolescence itself but the (social) environment the individual lives in during this phase which can bring about significant changes in its biobehavioural profile [17,21].

## Animals, materials & methods

### Animal husbandry

The experiments were carried out with ten male domestic guinea pigs (*Cavia aperea* f. *porcellus*) and eight male wild cavies (*Cavia aperea*), the wild ancestor of the guinea pig [10-13,33]. All individuals were kept in the Department of Behavioural Biology at the University of Münster.

The guinea pigs used for this study were male descendants of the department’s heterogeneous short-haired and multicoloured breeding stock of domestic guinea pigs. The wild cavies originated from a population that was also maintained at the department. This breeding stock was established in 1994 with individuals obtained from the University of Bayreuth and the University of Bielefeld. One year later, several wild born cavies were added to refresh the gene pool. As the founder cavies of the stock in Bayreuth and Bielefeld (established in 1985), these animals were caught in Argentina. In the following years, cavies from other laboratories were crossbred into the population to counteract inbreeding. Currently, the population has been bred for over 50 generations. The breeding protocol did not select for specific characteristics of the animals and was at random concerning selection of mating partners and aimed for outbreeding. A study comparing the population maintained at our laboratory for over 30 generations to wild-caught cavies and their first laboratory reared offspring showed that there are no differences between the two groups regarding basal cortisol and testosterone levels, cortisol reactivity, as well as a wide range of behaviour (sociopositive, orientation, male courtship, aggression, and exploration) [9].

Both, guinea pigs and cavies were born to single breeding pairs that lived in 0.5 × 1.0 m enclosures with a wall height of 0.5 m (guinea pigs) or 0.8 m (cavies). Food containing all relevant nutrients and water were available ad libitum and a cardboard box provided shelter. Guinea pigs could be identified by natural markings whereas cavies were marked individually by fur-coloration with 32% hydrogen peroxide since they possess a uniform brown/grey pelage that does not allow for individual differentiation.

Up to weaning each subject was kept together with its parents and litter mates. At the age of 20 (+1) days the subjects were removed from their parents’ enclosure and put together in pairs in a new housing enclosure (0.5 × 1.0 m; wall height: 0.5 m (guinea pigs)/0.8 m (cavies)). Never more than two brothers of each litter were used in the study and brothers were never housed together. Guinea pigs were housed with guinea pigs and cavies were housed with cavies. Age differences among the individuals of one experimental pair never exceeded 13 days. The older partner subject was temporarily (max 13 d) housed with a female until another male experimental animal of suitable age was available to complete the experimental pair. Temporary female housing partners were maximally eight days older than the subject they kept company with. Male-male pair housing is unproblematic in guinea pigs and cavies throughout adolescence. Dominance relationships in the housing groups were established without major aggression and were stable over the course of the experiment. In guinea pigs it was shown that dominance status of an animal does not interfere with performance in the tests used in the present study [28].

Both, guinea pigs and wild cavies were housed in the same husbandry room under standard conditions: 12:12 light-dark-cycle (lights on at 07:00 a.m.), temperature 22 ± 3°C, relative humidity 50% ± 10%. The bedding (wood shavings, Allspan Olympia-Einstreu, Allspan GmbH, Karlsruhe, Germany) was exchanged every two weeks. During cleaning the animals were removed from the enclosure and put into a cardboard box. Wild cavies were fed mixed pellet food at the rate of 2:1 (Höveler Meerschweinchenfutter 10700, Höveler Spezialfutterwerke GmbH & Co. KG, Dormagen, Germany and Altromin 3023, Altromin Spezialfutter GmbH & Co. KG, Lage, Germany). In addition, they received oatflakes (Fortin Mühlenwerke GmbH & Co. KG, Düsseldorf, Germany) once a week. Guinea pigs were only provided with commercial guinea pig diet (Höveler Meerschweinchenfutter 10700) since they tend to adiposity. Every week the cavy and guinea pig diet was supplemented once with straw and six times with hay. Twice a week the water bottles were cleaned and ascorbic acid (Altapharma Vitamin C Pulver, Dirk Rossmann GmbH, Burgwedel, Germany) was added to the fresh water. Several routine veterinary and animal health care procedures such as trimming the claws were executed throughout the whole experimental phase. All experiments were announced to the local authorities and were approved by the “Tierschutzbeauftragter” of the University of Münster (Reference number: 8.87-51.05.20.11.030). Experiments were carried out in accordance with the European Communities Council Directive of 24 November 1986 (86/609/EEC). All efforts were made to minimize animal suffering and to reduce the number of animals used.

### Experimental design

All animals went through two periods of testing which were performed during two specific periods of time in the life of the animals: early and late adolescence [22-24]. The first testing block started when the animals had reached an age of 51 (± 1) days and lasted up to an age of 62 (± 1) days. At this phase of life, male animals are already weaned for about 30 days and are becoming progressively more independent of their mother. They are, however, only on the brink of reaching sexual maturity which occurs around 75 days of age [29,30]. Hence, this testing phase is termed early adolescence. The second testing block took place when the individuals reached late adolescence (between 120 ± 1 d and 132 ± 1 d). At this age, animals are already fully sexually mature. In a housing situation with older, more dominant males present they are, however, not yet able to compete and gain dominant positions. Thus, they cannot yet gain access to receptive females in the late phase of adolescence. In a colony housing situation (guinea pigs), the social status that allows males to successfully defend and mate with females is reached at an age of six months at the earliest [35]. A similar situation is found in wild living cavy populations, in which only large, adult males have the ability to monopolise females [34].

During each testing block, animals were subjected to five behavioural tests and a stress reactivity test in the following order: *Open Field Test*, *Dark-Light Test*, *Step Down Test*, *Male vs. Infant Interaction Test*, *Male vs. Female Interaction Test*, and *Stress Reactivity Test*. Three major domains of guinea pig phenotypes (emotionality, social behaviour, and stress reactivity; [28]) can be assessed with these tests and thus they were used to characterise and compare the behavioural and stress reactivity (cortisol) profiles of guinea pigs and cavies. Since testosterone plays a crucial role in the maturation processes during adolescence, basal testosterone values were collected as well.

Open field, dark-light, and step down test were used to assess emotional behaviour. In contrast to our previous work [28], social behaviour was not only assessed in a *male vs. female interaction* test but also in a *male vs. infant interaction* test in the present study. Finally, the cortisol reactivity test was used to characterise the animals’ stress response when exposed to an unfamiliar environment [49]. Both testing phases lasted 13 days. Only one of the two housing partners of an experimental pair was tested on one day and animals only completed one test per day. Time between two subsequent tests never exceeded two days for each animal.

### Behavioural tests and stress reactivity test

All tests were conducted in a wooden enclosure of 1 × 1 m size with a wall height of 50 cm (guinea pigs) or 80 cm (cavies). The floor of the test enclosure was covered with wood shavings. In all behavioural tests the bedding was mixed with three handfuls of soiled shavings from the animal’s home enclosure in order to alleviate the aversive properties of the experiments. In the stress reactivity test only fresh bedding was used. For subsequent analysis all tests except for the stress reactivity test were videotaped. The experimenter was not present in the room during testing and the housing room of the experimental animals was not entered two hours prior to testing. Testing took place in a room other than the animals’ housing room. All behavioural tests lasted 15 min and were conducted between 13.00 h and 15.00 h. The stress reactivity test lasted four hours. The room in which the tests were conducted was illuminated by neon tubes. The lightning intensity in all tests was 290 ± 10 lx. Specific characteristics of the enclosure in the single tests are specified in the following. Behavioural scoring was done using focal animal sampling and continuous recording [50].

#### Tests on emotionality

Regarding emotional behaviour we conducted short behavioural tests that are commonly used to characterise emotional behaviour in mice and rats and adapted them for guinea pigs and cavies. These tests have already been successfully applied in guinea pigs [28] and comparable tests proved efficient in wild cavies [51].

##### Open field test

The 1 × 1 m test enclosure constituted the open field. Animals were carried to the testing room in a Makrolon type III cage filled with hay. The cage was put on the floor and the animals were allowed to acclimate for 60 s. The animals then were placed into the centre of the experimental enclosure. For analysis of explorative behaviour the enclosure was subdivided into 16 equal virtual squares (25 × 25 cm). The four inner squares were defined as the centre of the open field. Parameters recorded were: *virtual squares crossed* as a measure of exploration, and *percentage of time spent in the central area* as a measure of anxiety-like behaviour [52,53].

##### Dark-light test

The wooden dark-light box (300 mm width, 265 mm height, and 250 mm depth) was put at the midpoint of one of the enclosure’s walls. The top of the box could be opened for placement of the subject. The front of the box had a 150 × 300 mm door. The experimenter carried the animals from their home enclosure to the testing room in his arms and placed them into the closed dark-light box where they were allowed to acclimate for 60 s. Then, the front door was opened to allow the animal to explore the enclosure. Parameters recorded were: *latency to leave the dark box* and *percentage of time spent in the light area* as measures of anxiety-like behaviour as well as *number of times the animal entered the light area* as indicator of exploration [54]. Entering the enclosure was defined as the time point at which the animal completely moved out of the dark box into the enclosure.

##### Step down test

The step down tower was put in the centre of the enclosure, parallel to the walls. The tower had a base area of 300 × 300 mm and a height of 235 mm. The platform was covered with fresh wood shavings. Mounted 235 mm above the platform was a roof (300 × 300 mm). Animals were carried to the testing room in a Makrolon type III cage which was filled with hay. The cage was put on the floor and the animal was allowed to acclimate for 60 s. Subsequently it was placed on the platform facing one of the four edges. The animal was gently held still until its reflex to escape the experimenter ceased. The subject was then released, the experimenter left the room, and the *latency to step down* the platform was recorded as a measure of risk-taking. It was defined as the time point at which all four paws of the animal did not touch the platform anymore.

#### Social behaviour

##### Male vs. infant interaction

This test sought to investigate the general motivation of an animal to initiate social contact. To minimise the chance that the behaviour of the subject is driven by sexual or agonistic motivation, an unfamiliar same-sex infant was used as interaction partner (age 5 – 15 d). The infant was placed into the test enclosure under a small cage of bars to prevent approaches on the part of the infant. An identical but empty cage was also placed in the test enclosure as a control. The cages were ordinary chrome cutlery baskets (WENKO-WENSELAAR GmbH & Co. KG, Germany) turned upside down. They were 15 cm high and the square base of the openings was 169 cm^2^. Before testing the cutlery baskets were cleaned with 70% ethanol. They were positioned in two adjacent corners with 25 cm distance to the walls. An infant of the appropriate type (guinea pig or cavy) was put under one of the baskets. Subsequently the subject was carried to the testing room in a Makrolon type III cage filled with hay. The cage was put on the floor and after an acclimation period of 60 s the animal was placed into the enclosure. It was positioned against the middle of the wall opposite of the baskets with its head facing the baskets. Subsequently, the experimenter quietly left the room and the subject was allowed to make contact with the infant. After 15 min, first the subject and then the infant were placed back into their home enclosures. The following parameters were recorded: *latency to approach the unfamiliar infant* (subject touches the bars of the infant cage with its nose for the first time), and *duration of infant contact* (subject touches the bars of the infant cage with its nose). To exclude that the subject was only interested in the cage itself and not the infant inside it, the *duration of empty cage contact* (subject touches the bars of the empty cage with its nose) was compared to the *duration of infant contact*. Infants were only used once a day as an interaction partner and experimental animals were exposed to different infants during early and late adolescence.

##### Male vs. female interaction

For the male vs. female interaction test the enclosure was divided into two equal halves with a wooden board that was as high as the corresponding cage height (50 cm guinea pigs, 80 cm cavies). An unfamiliar female of the appropriate type (guinea pig or cavy) was put into one half. These females had given birth at least once, were pregnant and not due to give birth for the upcoming two weeks. Hence, females were never in oestrus during the experiments. No female was used more than once a day and subjects were never confronted to the same female in early and late adolescence. The experimental animal was carried to the testing room in the experimenter’s arms and was put in the other half of the enclosure. After an acclimation period of 60 s, the dividing wall was removed and the animals could explore each other. Behavioural patterns recorded were: *courtship behaviour* (*intensive anogenital licking*: subject lowers and turns its head and touches, sniffs, licks and/or nuzzles the anogenital region of the female; and *rumba*: subject rhythmically oscillates its hind quarters from side to side). These behavioural patterns were scored as frequencies. Furthermore the *latency to show courtship behaviour towards the unfamiliar female* was recorded. The behavioural definitions used are derived from previous studies on social behaviour in guinea pigs and cavies [33,35,55].

#### Stress reactivity test

Animals were placed into the centre of the enclosure that contained food and water ad libitum for 4 h. The floor was covered with fresh wood shavings. A novel environment has been shown to act as a psychological stressor in guinea pigs and cavies, causing an increase in cortisol (C) levels [4,49]. All tests were started at 13:00 h ± 15 min. At the beginning of each test the animal was caught from its home enclosure and a blood sample was taken to assess initial C (I) concentrations (for sampling method see below). After that, the animal was introduced into the unfamiliar environment. At 60 min (reaction value 1, R1), 120 min (R2), 180 min (R3), and 240 min (R4) subsequent blood samples were taken to determine changes in C concentrations. After the fifth blood sample, the animal was placed back into its home enclosure. The parameters evaluated were: I, *R1-R4, absolute C increase from I to R 1-4, and absolute C increase from I to the maximal reaction value* (MAX). To make sure that the exposure to the novel enclosure really induced a stress response, I-values were compared to the R values within guinea pigs and cavies. Furthermore, to compare cortisol-reactivity between guinea pigs and wild cavies, the I-values and R-values, the increases from I to all four R-values, and the increase from I to MAX were compared. Finally, development of I and R-values over adolescence was evaluated within guinea pigs and cavies.

Blood samples were collected from the blood vessels of the ears. A muscle salve (Elacur hot, Riemser Arzneimittel AG, Greifswald - Insel Riems, Germany) was applied to the ear to stimulate circulation and the vessels were illuminated with a cold-light lamp. Vessels were pricked with an injection needle and about 0.1 ml of blood was collected in heparinised capillary tubes. One experimenter held the animal in his/her lap, while a second collected the sample. All blood samples for determination of C levels were taken within the first three minutes after entering the room the animal was in. Plasma C levels do not increase significantly within the first five minutes after entering the room and hence the procedure reliably measured the plasma C levels the animals had before entering the room [29,56,57]. The blood sampling method used is a non-stressful procedure for the animals that does not elicit significant struggling [29,56]. Previous experiments showed that when placing the animals back to their familiar home enclosure after blood sampling, cortisol levels do not increase at all [29]. Hence, the sampling method itself does not have major impacts on the cortisol levels measured. Because no anaesthesia is required, hormone levels in the second, third, fourth, and fifth samples were not influenced by previous exposure to anaesthesia. Plasma was separated by centrifugation (11,700 × g for 7 min) and deep frozen (-20°C) until assayed.

Plasma C concentrations were determined using a luminescence enzyme-linked immunosorbent assay (ELISA; Cortisol ELISA Kit, IBL International GmbH, Hamburg, Germany). The antibody used cross-reacted with relevant steroids as follows: prednisolone 29.8%, 11-desoxycortisol 8.48%, cortisone 4.49%, prednisone 2.12%, corticosterone 1.99%, 6β-hydroxycortisol 1.03%. The intraassay %CV was 3.2%, the interassay %CV was 6.1%.

#### Plasma testosterone levels

The initial blood samples from the stress reactivity test were also used to determine basal plasma testosterone (T) levels. Plasma T concentrations were determined using a solid phase enzyme-linked immunosorbent assay (ELISA; Testosterone ELISA Kit, Demeditec Diagnostics GmbH, Kiel, Germany). The antibody used cross-reacted with relevant steroids as follows: 11 β-hydroxytestosterone 3.3%, 19-nortestosterone 3.3%, androstenedione 0.9%, 5 α-dihydrotestosterone 0.8%, 17 α-methyltestosterone, 0.1%, epitestosterone, oestradiol, progesterone, cortisol, oestrone, and danazol < 0.1% each. The intraassay %CV was 3.6%, the interassay %CV was 7.1%.

### Data analysis & statistics

VHS video tapes were digitised and subsequently evaluated using the behavioural observation and analysis program *The Observer XT* (Version 8, Noldus Information Technology, Wageningen, Netherlands). Sample sizes were relatively small in the present study (N_domestic_ = 10, N_wild_ = 8) and thus normal distribution of data could neither be assumed nor tested for. Consequently, non-parametric statistics were used. Due to technical difficulties, sample sizes are reduced in a few cases (see Results). To test for development of parameters over the course of adolescence in guinea pigs and cavies (from early to late adolescence) the Wilcoxon signed-rank test was used. To compare parameters between guinea pigs and cavies the Mann-Whitney U test was used. For comparison of k-related samples, the Friedman test was used with Kendall’s W to assess effect sizes. All calculations were done two-tailed. For all tests a significance-level (α) of 0.05 was selected. All tests were calculated using the software package IBM SPSS Statistics 20.0 (IBM Corporation).

## Competing interests

The authors declare that they have no competing interests.

## Authors’ contributions

BZ established the behavioural testing regimen used in the study (for guinea pigs), helped in conceiving of the study and its design, drafted the manuscript, analysed the data statistically, and created the figures. AS modified the behavioural testing regimen to the requirements of wild cavies, and acquired all data. SK and NS conceived of the study and its design, coordinated and supervised the whole study, and were involved in drafting the manuscript. All authors read and approved the final manuscript.
